# Circular RNAs Interaction with MiRNAs: Emerging Roles in Breast Cancer

**DOI:** 10.7150/ijms.62219

**Published:** 2021-07-11

**Authors:** Liu Gong, XiaoYun Zhou, Jiping Sun

**Affiliations:** Department of Medical Oncology, Hangzhou Xiasha Hospital, Hangzhou, Zhejiang Province, China

**Keywords:** Breast cancer, Circular RNAs, MiRNAs, Metastasis, Angiogenesis

## Abstract

Despite significant advances in cancer therapy strategies, breast cancer is one of the most common and lethal malignancies worldwide. Characterization of a new class of RNAs using next-generation sequencing opened new doors toward uncovering etiopathogenesis mechanisms of breast cancer as well as prognostic and diagnostic biomarkers. Circular RNAs (circRNAs) are a novel class of RNA with covalently closed and highly stable structures generated primarily from the back-splicing of precursor mRNAs. Although circRNAs exert their function through various mechanisms, acting as a sponge for miRNAs is their primary mechanism of function. Furthermore, growing evidence has shown that aberrant expression of circRNAs is involved in the various hallmarks of cancers. This paper reviews the biogenesis, characteristics, and mechanism of functions of circRNAs and their deregulation in various cancers. Finally, we focused on the circRNAs roles as a sponge for miRNAs in the development, metastasis, angiogenesis, drug resistance, apoptosis, and immune responses of breast cancer.

## Introduction

According to the GLOBOCAN 2018 estimates, breast cancer (BC) is the second most common cancer for both sexes (11.6% of the total diagnosed cancers) and the most common cancer among females 1. Despite advances in diagnostic and treatment, the 5-year survival rate for BC patients is 27.4% 2. Thus, developing more effective strategies for prophylactic intervention, prediction of prognosis, and therapy evaluation is crucial. BC is a highly heterogeneous malignancy with diverse intratumoral and intertumoral non-uniformity and wide variation in tumors among affected patients 3. BC has stratified based on the expression of progesterone receptor (PR), human epidermal growth factor receptor 2 (HER2), and estrogen receptor (ER) into four classes: luminal A (ER+, PR+, HER2-), luminal B (ER-, PR-, HER2±), HER2-positive (ER-, PR-, HER2+), and triple**-**negative (ER-, PR-, HER2-) 4. Using sequencing and modern genomic technologies, the molecular profile of BC has been widely deciphered, leading to provide novel information about dysregulated genes and transcripts. Hence, better classification of BC individuals based on the predictive biomarkers at the early stages and the molecular mechanism underlying BC tumorigenesis will help identify and treat BC patients.

Among dysregulated transcripts, non-coding RNAs (ncRNAs), including long-non-coding RNAs (lncRNAs), microRNAs (miRNAs), small nuclear RNAs (snRNAs), and circular RNAs (circRNAs), are associated with cancer development, progression, and treatment 5,6. The circRNAs, covalently closed RNA structures without 5′ caps or 3′ poly (A)-tails, are insensitive to degradation by RNases R; thus, they are more stable and abundant in the cell 7,8. It has been revealed that circRNAs are related to the pathogenesis of several human diseases, such as cancer. For instance, circRNAs can be involved in tumorigenesis and tumor progression by promoting self-sufficiency in growth signals, evading cell death, insensitivity anti-growth signals, limitless replication, sustained angiogenesis, and metastasis 9. Here, we outline the biogenesis and functional roles of circRNAs, describe dysregulation of circRNAs in BC, and the evidence for regulation of BC hallmarks by circRNAs during BC tumorigenesis, circRNAs potential as biomarkers, and their potential therapeutic targets.

## Biogenesis, degradation, and characteristics of circRNAs

Initially, Sanger *et al.* discovered circRNAs in RNA viroid in the 1970s 10. They were considered as by-products of pre-mRNA processing or the products of mis-splicing, because of the limitations of detection techniques, for a long time. However, abundant numbers of circRNAs in eukaryotes, including fungi, viruses, plants, and animals, have been identified owing to advances in bioinformatics and RNA high-throughput sequencing 11. As a result, several biochemical tools and techniques have been developed for the identification of circRNAs. Table 1 summarizes the developed tools for circRNAs identification and their advantages and disadvantages.

Following the progress of high-throughput transcriptome analysis techniques, various circRNAs have been predicted and recognized to exist at high levels and stably in body fluids, including serum, plasma, urine, and exosomes. The isolated circRNAs from body fluids and circulating cells contained in body fluids are identified and the analytical results are applied for diagnosis, progression, therapy selection, and therapy monitoring in different diseases, such as cancer 12. Due to the great variability in the length and genesis from liner RNAs, the isolation of circRNAs is not possible according to their size and/or sequence 13. Following whole RNA isolation, treatment with exoribonuclease, such as RNase R, is widely used for degrading linear RNAs to enrich circRNAs. Additional exonucleases may be helpful to isolate purer circRNAs. For instance, removing 5′ cap structures in mRNAs with RNA 5′ pyrophosphohydrolase (RppH) or tobacco acid pyrophosphatase (TAP) followed by digestion with an exonuclease degrades RNAs with 5′ cap 14.

**Figure 1:**
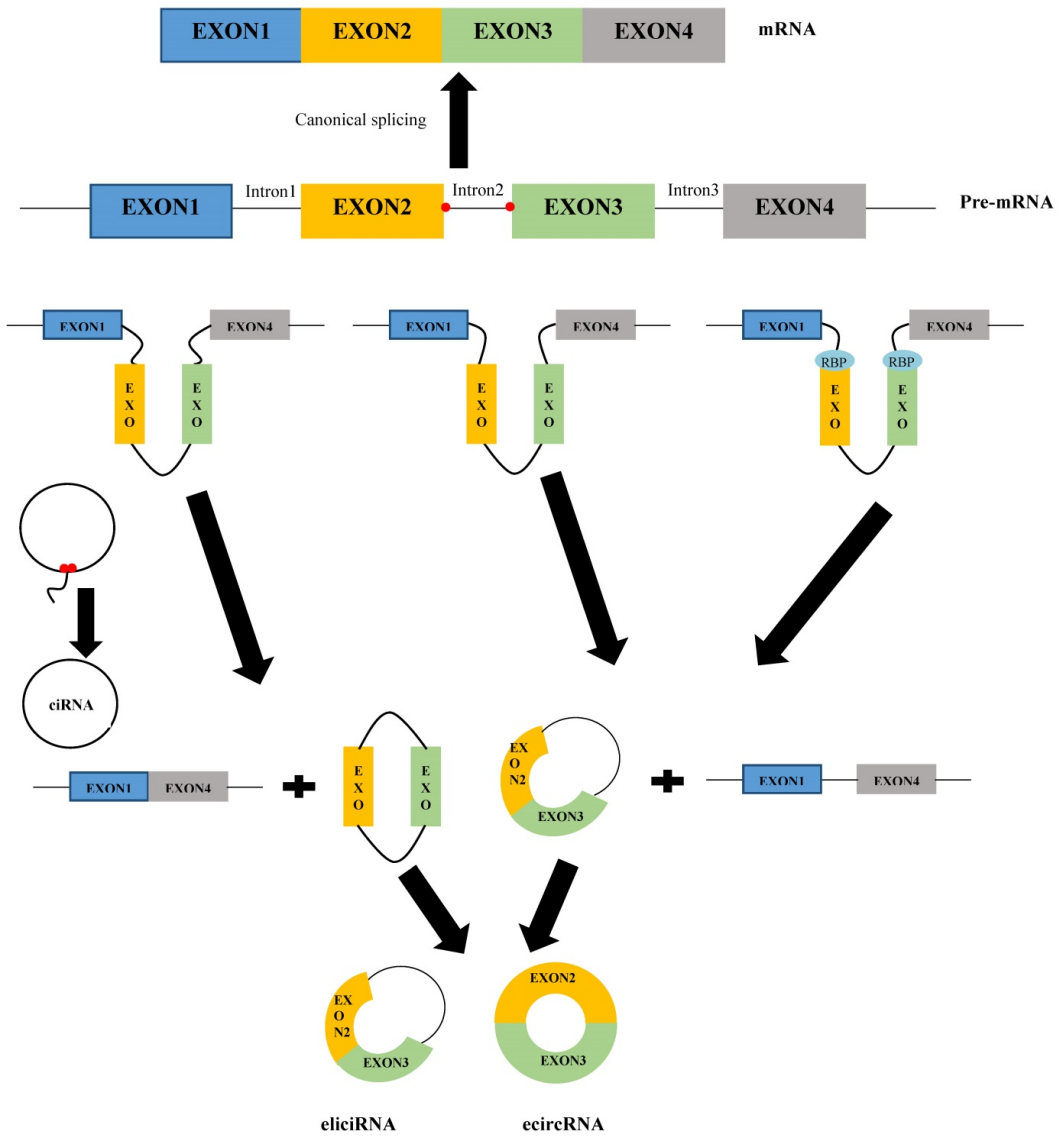
The mechanism of the formation of various types of circRNAs.

Similar to linear RNAs, circRNAs are derived from precursor mRNAs (pre-mRNAs). In contrast, linear RNAs are formed through classical splicing, while circRNAs usually are generated via back-splicing 15. Although a study revealed that 83% of circRNAs have overlap with exons 16, circRNAs can stem from all regions of the genome, including intronic, untranslational regions (UTRs), antisense, and intergenic 17. Based on their origin and mechanism of formation, circRNAs can be categorized into three classes: exonic circRNAs (ecircRNAs), circular intron or intronic circRNAs (ciRNAs), and exon-intron circRNAs (eiciRNAs) 18. Figure 1 shows the mechanism of the formation of various types of circRNAs. EcircRNAs, the most common circRNAs, are generated based on the three models: lariat-driven circularization (or exon skipping model), intron-pairing-driven circularization (or direct back splicing model), and RNA-binding proteins (RBPs)-driven circularization 19. In the lariat-driven circularization model, the 3′ end of the splice donor covalently joins to the 5′ end of the splice acceptor by exon skipping, leading to an exon-containing lariat structure and a linear mRNA. The introns of the lariat structure are removed by the spliceosome to form an exonic circRNA 12. In addition to the lariat-driven circularization model, ecircRNAs could be generated through another model called intron-pairing-driven circularization. In this model, the complementary motifs on the flanks of introns form circular and linear structures. In the circular structure, splicing out the introns mediates the formation of ecircRNAs 15,20. However, during the biogenesis of circRNAs, introns may not be removed completely but are retained between the circulated exons in the circRNA structure. This phenomenon leads to the formation of other types of circRNAs, so-called eiciRNAs 21. Furthermore, RBPs, including quaking (QKI), nuclear factor 90/110 (NF90/NF110), muscleblind (Mbl), DExH-box helicase 9 (DHX9), and adenosine deaminases that act on RNA 1 (ADAR1), also are involved in the biogenesis of ecircRNAs 22. The binding of RBPs to the flanking introns mediates circularization of single-stranded RNAs by bringing the flanking introns into the vicinity, leading to the formation of ecircRNAs or eiciRNAs 12,23. CiRNAs are formed when lariat introns escape from debranching and digestion. Their biogenesis depends on a 7 nt GU-rich element near the 5′ splice site and an 11 nt C-rich element near the branch-point site 24. The elements cause the intron to generate a circular structure at the branchpoint 2′-5′ junction. Finally, the formed lariats undergo 3′ tail degradation, leading to the formation of ciRNAs 24,25.

As mentioned later, one of the mechanisms that circRNAs exert their regulatory functions is their interaction with miRNAs in the cytoplasm 26,27. Although exosomal circRNAs also regulate cellular function by interacting with miRNAs, exosomes act as intercellular carriers of circRNAs and their regulatory functions occur in the cytoplasm of receptor cells 28,29. Several databases and software have been developed to predict circRNAs/miRNAs interaction and target mRNAs. For instance, the cancer-specific circRNA database (CSCD) at https://gb.whu.edu.cn/CSCD predicts RNA binding protein (RBP) and miRNA response element (MRE) sites 30. Also, the circular RNA Interactome (CircInteractome) web maps RBP and MRE sites on human circRNAs and provides binding sites on circRNAs 31.

To exert their function, the turnover of circRNAs and how they are degraded are crucial. Owing to 3′ poly (A)-tail and 5′ 7- methylguanosine cap in their structures, mRNAs are degraded by exonucleases shortening their length in 3′→5′ and 5′→3′ directions. Under certain conditions, some mRNAs are cleaved by endonucleases and then are degraded by the mentioned exonucleases. 32. Due to their circular structure and lack of 3′ poly (A)-tail and 5′ cap, circRNAs are cleaved by endonucleases. It has been demonstrated that circRNAs have a tendency to form imperfect duplexes and inhibit double-stranded RNA-activated protein kinase (PKR). Upon viral infection, RNase L endonuclease degrades circRNAs, leading to PKR activation and immune response induction 33. Under normal conditions, RBPs, UPF1, and G3BP1 selectively bind to highly-structured circRNAs regulate their decay 34. Furthermore, circRNAs carrying N6-methyladenosine (m6A) modification undergo endoribonucleolytic cleavage via ribonuclease P (RNase P)/mitochondrial RNA processing (MRP) complex in interaction with YTHDF2, which identifies m6A modification. In this cleavage system, heat-responsive protein 12 (HRSP12) acts as a bridge between the RNase P/MRP complex and YTHDF2 35.

In addition to their higher stability, there are several particular characteristics of circRNAs: (1) they have a length ranging from below 100 nts to larger than 1000 nts 36,37; (2) although most of the circRNAs are located in the cytoplasm (ecircRNAs), a small number resides in the nucleus (ciRNAs and eiciRNAs) 17,38; (3) they are broadly expressed in eukaryotic cells and more than one million circRNAs are detected in human 39; (4) the expression pattern of circRNAs has been considered as developmental stage-specific and cell type-specific 40; (5) the sequence of most of the circRNAs are conserved between different species 41; and (6) they play regulatory roles at both transcriptional and posttranscriptional levels 17.

## Functions of circRNA

**Figure 2:**
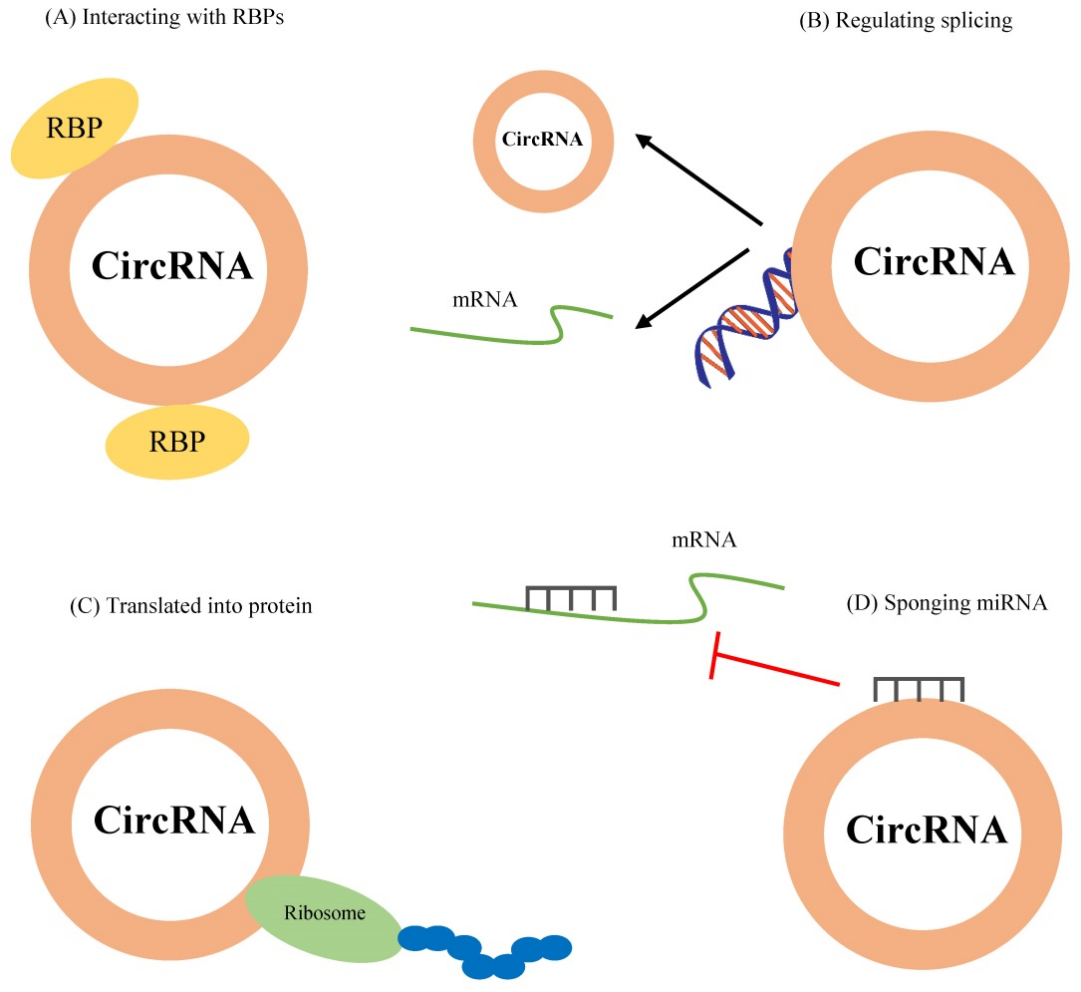
The function mechanisms of circRNAs. CircRNAs modulate cellular functions by (A) interacting with RBPs, (B) regulating splicing, (C) translation into proteins, and (D) sponging miRNAs.

Figure 2 represents the functional mechanisms of circRNAs in cells.

### Interaction with RBPs

Since RBPs are involved in various cellular processes, including proliferation, apoptosis, differentiation, senescence, migration, and oxidative stress responses, through posttranscriptional regulation of RNAs (transportation, splicing, and translation) 42, binding of RBPs to circRNAs and the formation of RNA-protein complexes (RPCs) can mediate several cellular functions. For instance, Zhu *et al.* found that circZKSCAN1 inhibits cancer stem cells in hepatocellular carcinoma via binding to the RBP fragile X mental retardation protein (FMRP). They showed that cell cycle and apoptosis regulator 1 (CCAR1), a coactivator of the Wnt/β-catenin pathway, is a downstream target of FMRP, suggesting this pathway as a potential therapeutic target 43. On the other hand, as mentioned above, RBPs mediate circRNAs formation. The RBP trinucleotide repeat-containing 6A (TNRC6A) regulates circ0006916 formation via binding to the intron regions 44.

### Regulation of transcription or splicing

It has been suggested that circRNAs, especially eiciRNAs and ciRNAs which are located in the nucleus, are involved in the regulation of gene transcription. For example, two eiciRNAs, circ-PAIP2 and circ-EIF3J, in association with the U1 snRNP can interact with RNA polymerase II to promote their parental genes' expression in HEK293 and HeLa cells, whereas their knockdown with either RNaseH-based antisense oligonucleotides or siRNAs decreases their parental gene expression 21. In another study, Zhang *et al.* indicated that the knockdown of ci-ankrd52, a ciRNA, with synthetic antisense targeting the pre-mRNA intron, suppresses mRNA levels of its parental gene 24.

### Translation into proteins

It has been reported that some ncRNAs contain small open reading frames (smORFs) for encoding small peptides which contribute to diverse cellular functions and processes, such as pathological conditions 45. Peptides or proteins encoded by ncRNA play crucial roles in cancer development and progression, including tumor cell metabolism and metastasis 46. Due to lacking internal ribosome entry sites (IRES), poly (A)-tail, and 5′-3′ polarity, circRNAs were primarily considered as non-coding RNAs 47,48. Besides their non-coding functions, convincing evidence revealed that circRNAs could be translated into proteins. For example, it has been shown that circ-ZNF609 has an open reading frame (ORF) and could translate into a protein that controls myoblast proliferation 49. Circ-FBXW7 is another circRNA that carries IRES and codes FBXW7-185aa protein. This protein acts as a tumor suppressor in glioma, which inhibits the proliferation of tumor cells 50. Mechanistically, the presence of the most common modified base of RNA structures within circRNA sequences, N6- methyladenosine(m6A), recruits YTHDF3 and eIF4G2, leading to translation initiation 51. However, only a small number of circRNAs have been identified to be involved in the translation.

### As miRNA sponges

Several studies have been demonstrated that circRNAs can act as competitiveendogenous RNAs (ceRNAs) via binding to miRNAs to suppress their function. MiRNAs are 21-25 nts in length non-coding RNAs that negatively regulate mRNA expression by binding to their 3′‐untranslated region 5. The most well-known circRNA in interaction with miRNAs is ciRS-7, also called CDR1as, which contains 63 conserved regions for binding to miR-7 17. Also, circSry acts as a sponge for miR-138 with having 16 conserved regions for binding to miR-138 40. Table 2 summarizes some circRNAs dysregulation in various cancers and their interaction with miRNAs, leading to upregulation of miRNA target mRNAs.

## CircRNAs as diagnostic and prognostic biomarkers in cancer

Owing to their stable structure, the long half-life, and the abundant presence in tissues and fluids, including plasma, blood, uterine, and exosomes, as well as expression in a developmental stage- and tissue-specific manner, circRNAs have been considered as promising biomarkers in the diagnosis and prognosis of various cancers 70.

The analysis of circSNAP47 expression in 83 tissue samples with qRT-PCR uncovered that circSNAP47 significantly upregulated in lung cancer patients. The higher expression levels of circSNAP47 were correlated with decreased overall survival (OS), advanced metastasis, and adverse prognosis 71. Similarly, upregulation of circ-ZKSCAN1 was closely related to poor prognosis, malignant characteristics, tumor stage, and tumor size in patients with lung cancer 72. In contrast, microarray and qRT-PCR analysis revealed that circBCAR3 upregulation in lung adenocarcinoma is correlated with improved OS, whereas its downregulation is associated with lymph node metastasis and advanced stage 73. It has been shown that higher expression of CDR1as is associated with poor prognosis and diagnosis of various cancers, including esophageal cancer, CRC, hepatocellular carcinoma, and gastric cancer 74-77. The analysis of circRNA expression in exosomes isolated from the serum of 170 CRC patients indicated that hsa-circ-0004771 distinguishes patients with stage I/II CRC and patients with benign intestinal diseases 78. In gastric cancer, the upregulation of circPRMT5, circLMTK2, and circHIPK3 is notably correlated with worse OS 79-81. Zou *et al.* identified circLARP4 as a prognosis biomarker in ovarian cancer which its lower expression was associated with poor prognosis in patients with ovarian cancer 82. Serum circRNA analysis revealed that the levels of circSETDB1 act as a predicting biomarker for response to chemotherapy in ovarian cancer 83. The summarize of circRNAs as prognostic and diagnostic biomarkers in various cancers is listed in Table 3.

## CircRNAs regulate the hallmarks of breast cancer in interaction with miRNAs

**Figure 3:**
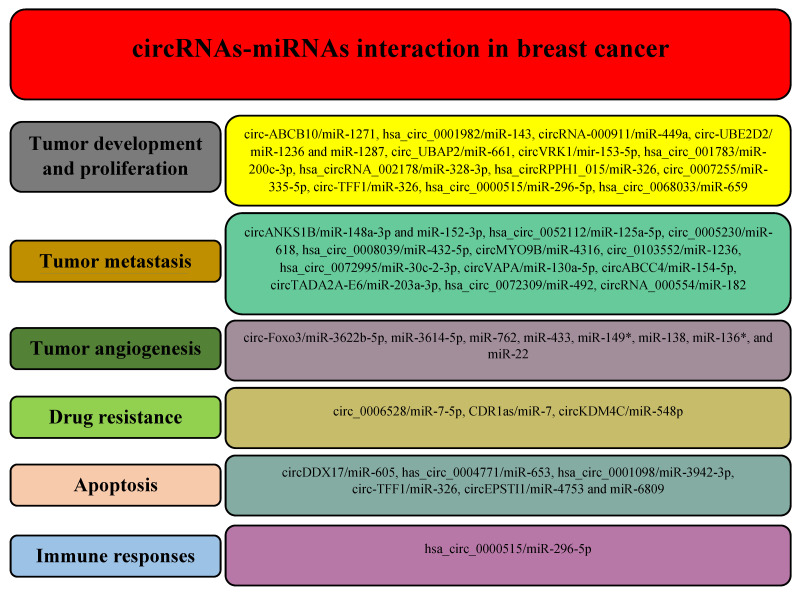
Different circRNAs regulate various hallmarks of breast cancer by modulating miRNAs.

Based on the mentioned techniques, a growing number of circRNAs have been identified with a potential role in the different processes during breast cancer, including carcinogenesis, metastasis, angiogenesis, response to treatments, apoptosis, and immune responses (Figure 3).

### CircRNAs and tumor development and proliferation

There is increasing evidence that circRNAs are involved in breast cancer development and proliferation. For example, circ-ABCB10 is significantly overexpressed in breast cancer cells and increases breast cancer cell proliferation and progression via directly targeting miR-1271 95. Similarly, hsa_circ_0001982 enhances carcinogenesis of breast cancer cells by negatively regulating miR-143, whereas knockdown of hsa_circ_0001982 inhibits cell proliferation and invasion and induces apoptosis 96. Microarray analysis manifested downregulation circRNA-000911 in breast cancer, whereas its upregulation promotes carcinogenesis via targeting miR‑449a, leading to elevation of Notch1 expression 97. In another study, Wang *et al.* demonstrated the upregulation of circ-UBE2D2 in breast cancer tissues was related to poor prognosis. They indicated that circ-UBE2D2 could promote breast cancer progression via acting as a sponge for two miRNAs, miR-1236 and miR-1287. They showed upregulation of MTA2, AFP, ZEB1, HOXB7, and KLF8, and downregulation of p21 expression as target genes of miR-1236, whereas the expression of CD105, PIK3CB, EGFR, GAGE1, ANGPT1, and ATF6α overexpressed as target genes of miR-1287. Therapeutically, intratumoral administration cholesterol-conjugated si-circ-UBE2D2 in a xenograft tumor model notably delayed tumor growth 98. circ_UBAP2 upregulation significantly promoted the progression of TNBC by modulating the miR-661/MTA1 pathway 99. Yan *et al.* found that circVRK1 is a tumor suppressor circRNA that suppresses the expansion and stemness of breast cancer cells through binding to mir-153-5p 100. It has been shown that mir-153 is involved in the maintenance of stemness of TNBC cells by targeting KLF5 101. There is evidence that cancer stem cells (CSCs) are the initiator cells of cancers and are responsible for cancer treatment failure owing to their pluripotency and self-renewal characteristics, leading to tumor recurrence 102,103. Other circRNAs also mediate breast cancer cell development and progression, including hsa_circ_001783, hsa_circRNA_002178, hsa_circRPPH1_015, circ_0007255, circ‐TFF1, hsa_circ_0000515, and hsa_circ_0068033, via sponging miR-200c-3p, miR-328-3p, miR-326, miR-335-5p, miR-326, miR-296-5p, and miR-659, respectively 104-110.

### CircRNAs and tumor metastasis

It has been demonstrated that metastasis is responsible for 90% of cancer-related deaths and is a primary determining factor in response to treatments 111. During the metastasis process, cancerous cells are dissociated from the primary site and systemically migrate through the vascular or lymphatic system to the secondary site. The migrated cells proliferate and survive in the secondary niche with the help of the components and cells within the tissue microenvironment 112. Epithelial to mesenchymal transition (EMT) plays an essential role in tumor metastasis in which non-motile epithelial cells are transformed into motile mesenchymal cells containing invasive properties 113.

There is growing evidence that circRNAs can mediate cancer metastasis. For example, circMTO1, hsa_circ_000984, circPIP5K1A, and circ-SMAD7 are associated with metastasis in bladder, colon, lung, and ovarian cancers 114-117. Yuan* et al.* found that circSCYL2 is downregulated in breast cancer cells, whereas its upregulation could inhibit the migration, invasion, and EMT progression in breast cancer cells 118. RNA analysis of TNBC tissues and cell lines revealed that circANKS1B is overexpressed in TNBC, leading to the promotion of breast cancer cell migration, invasion, and metastasis both *in vitro* and *in vivo* via inducing EMT. Mechanistically, circANKS1B acts as a sponge for two miRNAs, miR-148a-3p and miR-152-3p, which leads to upregulation of their target, upstream transcription factor 1 gene (USF1). Upregulation of the USF1 transcription factor transcriptionally increases the expression of TGF-β1, resulting in activation of the TGF-β1/Smad pathway to stimulate EMT 119. In another study, Zhang *et al.* found that hsa_circ_0052112 could promote migration and invasion of MCF-7 cells via directly sponging miR-125a-5p and regulating ZNF83 expression, whereas upregulation of miR-125a-5p notably reduces cell migration and invasion 120. Circ_0005230 is another pro-metastatic circRNA in breast cancer that promotes cell invasion by sponging miR-618 and regulating the expression of chromobox protein homolog 8 (CBX8) 121. It has been reported that CBX8 is an oncogenic protein that promotes the migratory properties of several cancer cells 122. Using microarray and qRT-PCR analyses, Liu *et al.* identified the overexpression of hsa_circ_0008039 in breast cancer samples which increases cell proliferation and migration via sponging miR-432-5p and elevating E2F3 expression 123. E2F3 is an oncogene that enhances tumor growth and metastasis in various cancers 124-126. Wang *et al.* indicated that circMYO9B is upregulated in breast cancer tissues, whereas its knockdown remarkably inhibits cell proliferation and invasion *in vitro* and tumor growth *in vivo*. Mechanistically, circMYO9B enhances the expression of FOXP4 through directly sponging miR-4316 127. By microarray, Du *et al.* found that circSKA3 is highly expressed in breast cancer cells and promoted cell invasion. The pro-metastatic property of circSKA3 is correlated with its binding to Tks5 and integrin β1, which promotes invadopodia formation 128. Invadopodia are actin, integrins, and TKS5 rich structures on the cancer cell membrane's outer surface that facilitate cancer cell movement and invasiveness 129,130. In addition to the mentioned circRNAs, there are other ones that promote migratory properties and metastasis of breast cancer cells, including circ_0103552, hsa_circ_0072995, circVAPA, and circABCC4, which act as a sponge for miR‐1236, miR-30c-2-3p, miR-130a-5p, and miR-154-5p, respectively 131-134.

There are also circRNAs that act as suppressors and negatively regulate the metastasis of breast cancer. For instance, Xu *et al.* indicated that two circTADA2As, circTADA2A-E6 and circTADA2A-E5/E6, are suppressors of progression and metastasis of breast cancer. The expression levels of these two circRNAs were significantly reduced in breast cancer patients and their downregulation was associated with poor survival of patients with TNBC. They showed that circTADA2A-E6 could exert their inhibitory effects on metastasis via targeting miR-203a-3p and restoring SOCS3 expression 135. It has been demonstrated that SOCS3 has anti-proliferative and anti-metastatic properties in breast cancer 136,137. In another study, Yan *et al.* identified hsa_circ_0072309 as another suppressor circRNA that inhibits breast cancer cell proliferation and invasion via inhibiting miR-492 138. In silico and RT‐qPCR analyses demonstrated that circRNA_000554 is notably reduced in breast cancer tissues and cell lines. Transfection of breast cancer cells with circRNA_000554 suppresses cell migration and invasion via reversing the EMT process and sponging miR‐182 139. Zinc finger protein 36 (ZFP36) is the direct target of miR-182 139 that acts as a tumor suppressor gene by inhibiting tumor cell proliferation and growth and inducing cell cycle arrest 140. Hou *et al.* reported that circASS1 expression is downregulated in MDA-MB-231, whereas its upregulation inhibits the invasiveness of breast cancer cells 141.

### CircRNAs and tumor angiogenesis

To proliferate and survive, tumor cells need blood vessel formation to supply sufficient nutrients and oxygen, so-called angiogenesis 142,143. This process requires pro-angiogenic factors, including vascular endothelial growth factors (VEGFs), platelet-derived growth factors (PDGFs), fibroblast growth factors (FGFs), hypoxia-inducible factors (HIFs), transforming growth factors (TGFs), and some chemokines 144. VEGF is the most potent promoter among the angiogenic agents, which exerts its functions through VEGF receptors (VEGFRs) 145. Mechanistically, the binding of pro-angiogenic factors to their cognate receptors leads to the production of proteases, such as matrix metalloproteinases (MMPs), which degrade extracellular matrix components. Then, endothelial cells (ECs) proliferate and migrate into the degraded area to form primary sprouts. The lamination of primary sprouts results in vascular tube formation and subsequent maturation to blood vessels 146.

It has been demonstrated that various circRNAs are associated with tumor angiogenesis in different cancers, including circ-001971, circRNA-MYLK, circ-RanGAP1, and circSMARCA5 in the colorectal, bladder, and gastric cancers, and glioblastoma, respectively 69,147-149. In a study, Yang *et al.* investigated the effect of two non-coding RNAs, Foxo3 pseudogene (Foxo3P) and circ-Foxo3, on the proliferation and angiogenesis of breast cancer cells. They revealed that non-cancerous cell lines express high levels of Foxo3, Foxo3P, and circ-Foxo3 compared to cancer cells, whereas transfection of Foxo3, Foxo3P, and circ-Foxo3 into MDA-MB-231 cells, triple-negative breast cancer (TNBC) cells, significantly decreased proliferation and survival of cancer cells. Furthermore, subcutaneous injection of circ-Foxo3-, Foxo3-, Foxo3P-transfected MDA-MB-231 cells into nude mice indicated that tumor growth in mice receiving transfected cells was notably slower than those received the control cells. They demonstrated that the inhibitory effects of Foxo3, Foxo3P, and circ-Foxo3 on tumor growth were due to decreased blood vessel formation and nutrition supply. Also, they showed that breast cancer cells transfected with Foxo3, Foxo3P, and circ-Foxo3 underwent extensive apoptosis. To exert their anti-tumor effects, both Foxo3P and circ-Foxo3 acted as a sponge for eight miRNAs, including miR-3622b-5p, miR-3614-5p, miR-762, miR-433, miR-149*, miR-138, miR-136*, and miR-22. Sponging these miRNAs increased free Foxo3 mRNAs and promoted their translation 150. It has been shown that Foxo3 upregulation increases apoptosis and inhibits angiogenesis by downregulating an antiapoptotic protein, FLIP, and decreasing EC proliferation, migration, and tube formation 151-153.

### CircRNAs and drug resistance

**Figure 4:**
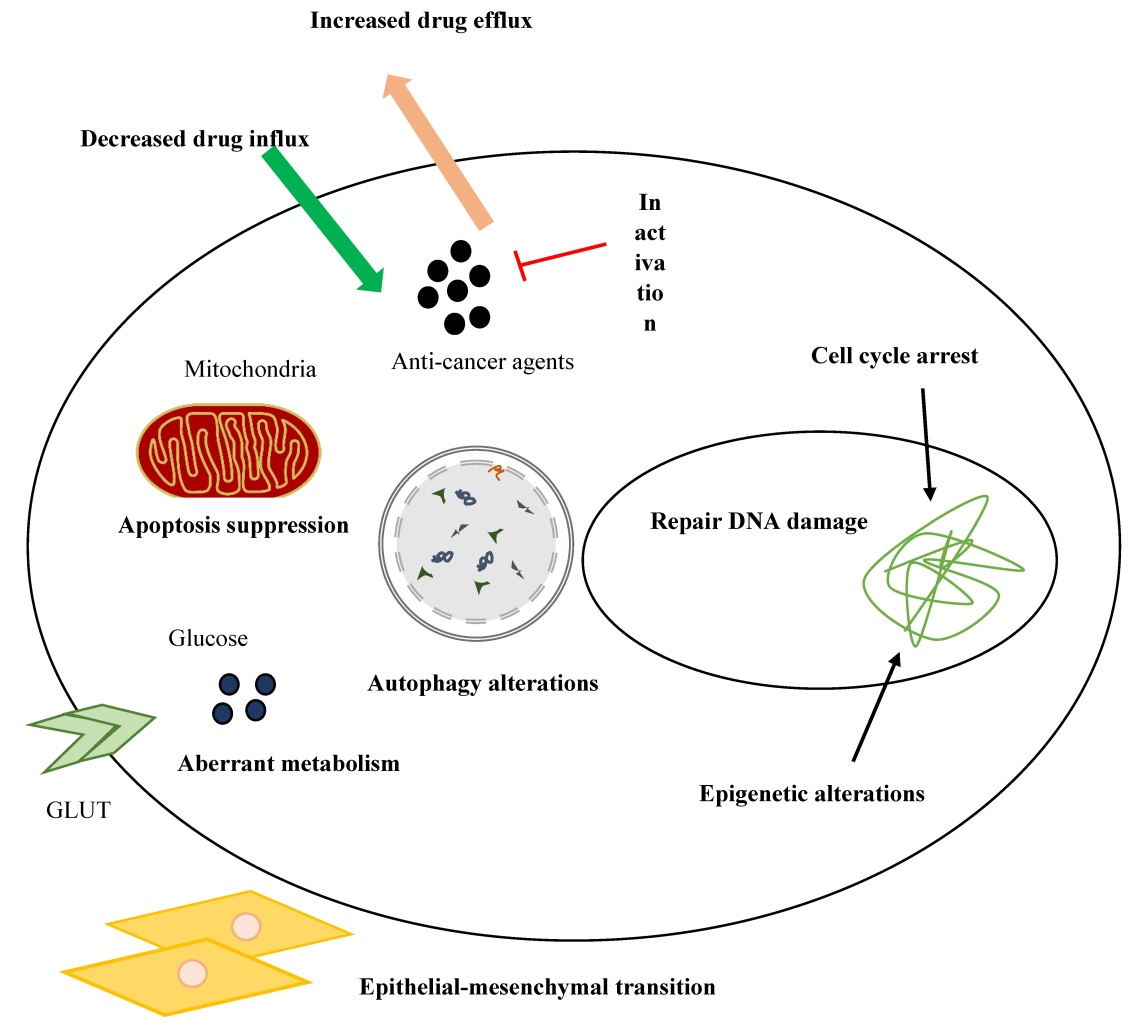
Drug resistance mechanisms in cancer cells.

Despite significant advances in the therapeutic strategies of breast cancer, resistance to treatments is a major obstacle to successful cancer therapy. Several mechanisms have been identified in drug resistance of breast cancer, including dysregulation of ATP binding cassette (ABC) transporters, modifications of signaling pathways, EMT and CSCs, cell cycle arrest, autophagy, and apoptosis 154. Figure 4 represents drug resistance mechanisms in cancer cells.

It has been shown that circRNAs are involved in resistance to chemotherapy. For example, circPVT1 upregulation mediates resistance of osteosarcoma cells to cisplatin and doxorubicin via regulating ABCB1, whereas knockdown of circPVT1 enhances chemoresistance of the cancer cells 91. Moreover, a number of circRNAs are involved in the resistance of breast cancer cells to therapeutic agents. The comparison of circRNA expression between monastrol resistant and non-resistance cells with microarray analysis by Liu *et al.* revealed that 398 circRNAs were dysregulated between the cell types. Further analyses demonstrated that the expression levels of hsa‑circRNA-007874 (circRNA‑MTO1) were decreased in monastrol-resistant breast cancer cells, whereas its upregulation enhanced the sensitivity of the cells to monastrol and inhibited cell viability. CircRNA‑MTO1 exerts its anti-tumor functions by targeting Eg5 protein without influencing Eg5 at the mRNA level. Mechanistically, circRNA‑MTO1 acts as a ceRNA for tumor necrosis factor receptor-associated factor 4 (TRAF4) which inhibits its interaction with the Eg5 gene, resulting in suppression of Eg5 at the protein level 155. TRAF4 is an oncogene that promotes translation without affecting the mRNA level via binding to the 3′ UTR of the target gene 156. In another study, Ma *et al.* identified circAMOTL1 as a mediator of paclitaxel resistance in MDA-MB-231 cells. On the other hand, siRNA against circAMOTL1 reversed the resistance in breast cancer cells by regulating the AKT pathway 157. Gao *et al.* revealed that among 3093 detected circRNAs, 18 circRNAs are differentially expressed between adriamycin (ADM) resistant MCF-7 cells (MCF-7/ADM) and parental MCF-7 cells. They identified that circ_0006528 is highly expressed in ADM-resistant tissues and cell lines compared to ADM-sensitive ones, whereas circ_0006528 knockdown using a siRNA increased the sensitivity of breast cancer cells to ADM. Mechanistically, circ_0006528 targets miR-7-5p which leads to overexpression of Raf1 158. It has been shown that Raf1 protein is involved in the drug resistance of breast cancer cells via regulating the ERK pathway 159. In another study, Yang *et al.* investigated the expression and role of CDR1as in the resistance of breast cancer cells to chemotherapy with 5‐fluorouracil (5‐FU). They uncovered the overexpression of CDR1as and downregulation of miR-7 in 5‐FU-resistant breast cancer cells, whereas knockdown of CDR1as or ectopic overexpression of miR-7 increased chemosensitivity of the cells by promoting apoptosis 160. circKDM4C is another circRNA that plays a role in the chemoresistance of breast cancer. The expression levels of circKDM4C are decreased in breast cancer and its low levels are associated with metastasis and poor prognosis. CircKDM4C could attenuate doxorubicin resistance of breast cancer cells via targeting the miR-548p/ phenazine biosynthesis-like domain-containing protein (PBLD) axis 161.

### CircRNAs and apoptosis

Apoptosis, programmed cell death, is a highly selective and crucial process that balances cell survival and death to prevent various diseases, including cancer 162. Generally, there are two pathways of apoptosis: extrinsic (death receptor) and intrinsic (mitochondrial). In the extrinsic pathway, binding of extracellular ligands, such as Fas ligand (Fas-L), tumor necrosis factor (TNF), and TNF-related apoptosis-inducing ligand (TRAIL) to their cognate receptors initiates death signals, whereas the intrinsic pathway is activated after an increase of mitochondrial permeability and release of pro-apoptotic proteins 163. In both of the pathways, cysteine proteases called caspases are activated and cleaved the proteins.

Recently, Peng *et al.* revealed that circDDX17 levels were decreased in breast cancer, whereas its upregulation promoted cell apoptosis and reduced cell proliferation and colony formation through the regulation of p21 and CDK genes via directly binding to miR-605 164. High-throughput RNA sequencing revealed that 49 circRNAs were differentially expressed (30 were upregulated, and 19 were downregulated) between breast cancer tissues and nontumorous ones. Further analyses indicated that has_circ_0004771 expression was higher than other circRNAs in breast cancer tissues. Has_circ_0004771 upregulated the expression of Zinc finger E-box binding homeobox 2 (ZEB2) by binding to miR-653. Has_circ_0004771 and ZEB2 knockdown could induce apoptosis and inhibition of cell growth 165. In another study, Zhao *et al.* found that hsa_circ_0001098 (circRNA_BARD1) overexpression could promote breast cancer cell apoptosis by regulating the miR-3942-3p/BARD1 axis 166. It has been demonstrated that BARD1could promote apoptosis through binding to, phosphorylating, and stabilizing p53 in DNA double-strand break repair 167. In addition to the mentioned circRNAs, it has been shown that circ‐TFF1 (hsa_circ_0061825) and circEPSTI1 also are involved in the apoptosis of breast cancer cells through sponging miRNAs. Silencing circ‐TFF1 promotes apoptosis by miR‐326/TFF1 axis 108, while circEPSTI1 knockdown induces apoptosis via regulating the miR-4753 and miR-6809/ BCL11A pathway 168.

### CircRNAs and immune responses

According to the cancer immunosurveillance concept, tumor cells are identified, eradicated, and repressed by host immune systems 169. However, tumor cells can escape from immunosurveillance and immune control, resulting in tumor proliferation and clinical emergence 170. Among immune cells, myeloid-derived suppressor cells (MDSCs), regulatory T (Treg) cells, and tumor-associated macrophages (TAMs) act as immunosuppressive cells, whereas cytotoxic T cells (CTLs), T helper (Th) lymphocytes, and natural killer (NK) cells fight against cancer 171. Furthermore, inhibitory proteins, including programmed death receptor-1 (PD-1) and cytotoxic T-lymphocyte-associated antigen-4 (CTLA-4), also mediate immune evasion of cancer cells. Thus, blocking the inhibitory proteins has been developed as a promising strategy in breast cancer therapy 172,173.

It has been shown that various circRNAs are involved in the immune regulation of cancers 174. For instance, Zhang *et al.* found that upregulation of hsa_circ_0020397 in colorectal cancer mediates cell viability and invasion via targeting miR‐138, leading to overexpression of PD‐L1 and telomerase reverse transcriptase (TERT) 175. Circ‐UBAP2 is another circRNA that promotes cancer progression by enhancing immune evasion. It was demonstrated that the higher levels of circ‐UBAP2 modulate the ZEB1 and CXCR4 expression in pancreatic adenocarcinoma, which is correlated with exhausted T cells, Tregs, and M2 macrophages as well as higher levels of PD-1 and CTLA-4 176. Cai *et al.* indicated that upregulation of hsa_circ_0000515 in breast cancer was associated with poor prognosis, whereas its silencing impaired cell proliferation, the progression of the cell cycle, and invasion, reduced pro-angiogenic potential, and decreased inflammatory response of breast cancer. Mechanistically, hsa_circ_0000515 acts as a sponge for miR‐296‐5p to inhibit CXCL10 repression 109. The binding of CXCL10 to the CXCR3 receptor activates and initiates G protein‐mediated signaling, resulting in Th1 cell generation and their aggregation at the inflammatory niche 177. Moreover, CXCL10 acts as an oncogene that is associated with breast cancer progression 177.

## Conclusions and perspectives

There is growing evidence that the circRNAs expression can affect breast cancer development and progression and may show great potential as a biomarker in diagnosing, prognosis, and treatment of breast cancer. Compared to other RNAs, circRNAs contain a stable structure and are broadly expressed in body fluids such as blood and uterine, suggesting circRNAs as promising and reliable biomarkers. Moreover, circRNAs detection using qRT-PCR and RNA sequencing is more accurate and convenient and needs a shorter time and smaller amounts of samples than the detection of proteins. However, their biological functions, formation, maturation, and transport from the nucleus to the cytoplasm need further investigation. Due to our incomplete understanding of circRNAs, some issues need to be addressed for their application as diagnostic and therapeutic biomarkers as well as therapeutic agents in clinical practice. First, it is necessary to assess the specificity and sensitivity of circRNAs as potential biomarkers. Second, targeting a circRNA as a therapeutic strategy for cancer therapy has potential risks for off-target effects on other tissues because a circRNA participates in the function of many tissues. Third, selecting an efficient delivery system, including viral vectors, liposomes, nanoparticles, and exosomes, based on the target cells is essential for the delivery of large amounts of circRNA. Another strategy to enhance their efficacy is engineering artificial circRNAs containing a large number of binding sites for miRNAs. Thus, circRNAs can be used in combination with other prognostic and diagnostic biomarkers for breast cancer, and their clinical application still requires more analysis and optimization.

## Author Contributions

LG provided suggestions and edited the manuscript. XYZ and JS drafted the manuscript.

## Figures and Tables

**Table 1:** Advantages and disadvantages of different tools in the identification of circRNAs.

Tools/Techniques	Advantages	Disadvantages
Reverse transcription-PCR (RT-PCR)	1. Simple 2. Rapid	1. Has biases 2. Has artifactual 3. Requires RNase R to distinguish from linear isoforms 4. Low-throughput
Northern blotting	1. Simple 2. No need to RNase R	1. Time-consuming 2. Laborious
Microarray	1. High-throughput 2. Sensitive 3. Managable in size	1. Only detects the known circRNAs 2. Identifies only based on junction sequences 3. Higher noise due to mismatch between probe and target
RNA sequencing (RNA-seq)	1. Detects novel circRNAs 2. More reliable and accurate	1. Low detection efficiency due to limitation in the detection of head-to-tail junctions 2. Highly expensive

**Table 2:** CircRNAs act as sponges for miRNAs in various cancers.

CircRNA	Up/Downregulated	Cancer	Target	Ref
circDLGAP4	Upregulated	Lung	miR-143/CDK1	52
circ-0006282	Upregulated	Gastric	miR-155/ FBXO22	53
circBCRC-3	Downregulated	Bladder	miR-182-5p/p27	54
cSMARCA5	Upregulated	Cervical	miR‑432/ERK	55
circZFR	Upregulated	PTC	miR-1261/C8orf4	56
circMCTP2	Downregulated	Gastric	miR-99a-5p/MTMR3	57
circATRNL1	Downregulated	OSCC	miR-23a-3p/PTEN	58
circ-CPA4	Upregulated	Lung	let-7 miRNA/PD-L1	59
circ-ITCH	Downregulated	Cervical	miR-93-5p/FOXK2	60
circPVT1	Upregulated	HCC	miR‐3666/SIRT7	61
circFAM114A2	Downregulated	Bladder	miR-762/∆NP63	62
hsa_circ_0053277	Upregulated	CRC	miR‐2467‐3p/MMP14	63
circEXOC6B	Downregulated	Ovarian	miR-421/RSU1	64
circ_SFMBT2	Downregulated	Glioma	mir-182-5p/Mtss1	65
hsa_circ_0000370	Upregulated	AML	miR-1299/ S100A7A	66
circMTO1	Downregulated	HCC	miR‐9/p21	67
circ_0020710	Upregulated	Melanoma	miR-370-3p/CXCL12	68
circ-001971	Upregulated	CRC	miR-29c-3p/VEGFA	69

CDK1, cyclin-dependent kinase 1; FBXO22, f-box protein 22; PTC, papillary thyroid cancer; MTMR3, myotubularin-related protein 3; OSCC, oral squamous cell carcinoma; PD-L1, programmed cell death ligand 1; FOXK2, forkhead box K2; HCC, hepatocellular carcinoma; SIRT7, Sirtuin 7; CRC, colorectal cancer; MMP14, matrix metalloproteinase 14; RSU1, ras suppressor-1; Mtss1, metastasis suppressor 1; AML, acute myeloid leukemia; VEGFA, vascular endothelial growth factor A.

**Table 3:** CircRNAs as prognostic and diagnostic biomarkers in various cancers.

CircRNA	Cancer	Prognostic/Diagnostic	Source	Method	Ref
circ-ITCH	Lung	Diagnostic	Tissue	RT	84
circMTO1	HCC	Diagnostic	Tissue	MA/RT	67
circPVT1	Gastric	Prognostic/Diagnostic	Tissue	NGS/RT	85
circ_0034642	Glioma	Prognostic	Tissue	RT	86
hsa_circ_0014717	CRC	Prognostic/Diagnostic	Tissue	RT	87
hsa_circ_002059	Gastric	Prognostic/Diagnostic	Tissue/Plasma	RT	41
has_circ_0067934	ESCC	Prognostic	Tissue	RT	88
circ-LDLRAD3	Pancreatic	Diagnostic	Tissue/Plasma	RT	89
hsa_circ_0126897	CRC	Diagnostic	Tissue	MA	90
circPVT1	OS	Diagnostic	Tissue/Serum	RT	91
circFARSA	Lung	Diagnostic	Tissue/Exosome	NGS/RT	92
circ-PDE8A	Pancreatic	Prognostic	Exosome	MA	29
CDR1as	HCC	Diagnostic	Tissue	RT	93
circLPAR1	Bladder	Prognostic	Tissue	RT	94

RT, qRT-PCR; HCC, hepatocellular carcinoma; MA, microarray; NGS, next-generation sequencing (RNAseq); CRC, colorectal cancer; ESCC, esophageal squamous cell carcinoma; OS, osteosarcoma.

## Abbreviations

BC: breast cancer
HER2: human epidermal growth factor receptor 2
miRNAs: microRNAs
circRNAs: circular RNAs
ecircRNAs: exonic circRNAs
ciRNAs: circular intron or intronic circRNAs
eiciRNAs: exon-intron circRNAs
RBPs: RNA-binding proteins
ceRNAs: competitive endogenous RNAs
OS: overall survival
EMT: epithelial to mesenchymal transition
TNBC: triple-negative breast cancer
PD-1: programmed death receptor-1
CTLA-4: cytotoxic T-lymphocyte-associated antigen-4
